# A critical discourse analysis of adolescent fertility in Zambia: a postcolonial perspective

**DOI:** 10.1186/s12978-021-01093-z

**Published:** 2021-04-06

**Authors:** Margarate N. Munakampe, Charles Michelo, Joseph M. Zulu

**Affiliations:** 1 Department of Health Policy and Management, School of Public Health, The University of Zambia, Lusaka, Zambia; 2 Department of Epidemiology & Biostatistics, School of Public Health, The University of Zambia, Lusaka, Zambia; 3 Strategic Centre for Health Systems Metrics & Evaluations (SCHEME), Department of Epidemiology & Biostatistics, School of Public Health, University of Zambia, Lusaka, Zambia

**Keywords:** Adolescent fertility, Double consciousness, Sexual and reproductive health, Postcolonial criticism

## Abstract

**Background:**

Despite global and regional policies that promote the reduction of adolescent fertility through ending early marriages and reducing early child-bearing, adolescent fertility remains high in most sub-Saharan countries. This study aimed to explore the competing discourses that shape adolescent fertility control in Zambia.

**Methods:**

A qualitative case study design was adopted, involving 33 individual interviews and 9 focus group discussions with adolescents and other key-informants such as parents, teachers and policymakers. Thematic and critical discourse analysis were used to analyze the data.

**Results:**

Adolescents’ age significantly reduced their access to Sexual and Reproductive Health, SRH services. Also, adolescent fertility discussions were influenced by marital norms and Christian beliefs, as well as health and rights values. While early marriage or child-bearing was discouraged, married adolescents and adolescents who had given birth before faced fewer challenges when accessing SRH information and services compared to their unmarried or nulliparous counterparts. Besides, the major influencers such as parents, teachers and health workers were also conflicted about how to package SRH information to young people, due to their varying roles in the community.

**Conclusion:**

The pluralistic view of adolescent fertility is fueled by “multiple consciousnesses”. This is evidenced by the divergent discourses that shape adolescent fertility control in Zambia, compounded by the disempowered position of adolescents in their communities. We assert that the competing moral worlds, correct in their own right, viewed within the historical and social context unearth significant barriers to the success of interventions targeted towards adolescents’ fertility control in Zambia, thereby propagating the growing problem of high adolescent fertility. This suggests proactive consideration of these discourses when designing and implementing adolescent fertility interventions.

**Supplementary Information:**

The online version contains supplementary material available at 10.1186/s12978-021-01093-z.

## Plain English summary

Global efforts have attempted to reduce the high adolescent fertility through various interventions. Early child bearing and marriage continue to fuel high adolescent fertility rates in low income settings, resulting in consequences such as mortality, morbidity, unsafe abortions and low birth weight. Using focus group discussions and interviews with young people, community members and policy makers, this qualitative study aimed to explore the different discourses that surround adolescent fertility in Zambia. We identified the influence of the age of adolescents, marital status and Christian values on adolescent fertility discussions. In addition, various influencers of adolescent fertility-related decisions-their parents, teachers, health workers and friends, faced difficulty with which sort of information to package for young people, since they also shared conflicting views too. We suggest that packaging messages for young people that are the same across different influencers and increasing their freedom to make informed decisions would help to reduce the soaring fertility rates among adolescents.

## Background

Globally, there are 1.2 billion adolescents aged 10–19 years [1]. Approximately 90% of adolescents live in low and middle-income countries (LMICs), which are also burdened with high adolescent fertility rates [1–4]. While 90% of adolescent births among 15–19 year olds occur within marriage, the lowest economic quintile reports substantially higher birth rates than the highest quintile [1]. Since the International Conference on Population and Development (ICPD) at Cairo in 1994, adolescent sexual and reproductive health (SRH) has risen to the development agenda, with adolescent fertility noted as a major priority [5]. Adolescent fertility, defined as ‘any pregnancy-related experience among those 10–19 years of age, including live birth, abortion, stillbirth, or miscarriage’ [6] thrives in low resource settings, usually driven by low educational attainment and early-marriages [7].

Despite efforts made to curb its’ drivers, adolescent fertility remains high in Zambia, with teenage pregnancy consistently high over the past 15 years [8–10]. About 29% of adolescent 15–19 have already given birth- 6% of the 15-year-olds and 58% of those aged 19 [8]. About 42% adolescents with no education, 46% of adolescents in the lowest wealth quintile and 73% of married adolescents had given birth before [8, 11]. Almost 30% of girls were married by the time they reached 18 years old [12]. Despite a global and regional push towards ending child marriage and early childbearing [12], a key intervention in the Sustainable Development Agenda, and with steady progress made towards increasing access to adolescent-friendly health services[13], most low-resource settings, including Zambia, regard the majority of the teenage pregnancies within marriage, but share negative attitudes towards pregnancies out of wedlock [14]. Disproportionate access to contraception and safe abortion services among adolescents has also been tied to marital status and other factors related to low socio-economic status [7, 13].

Several contextual factors such as traditional marital norms and Christian values—particularly in Zambia, a nation constitutionally declared Christian, play a restrictive role in shaping adolescent fertility control and ordinarily opposing the global health view of fertility control which is rights-based, regardless of the user [12, 15, 16]. The two divergent views: the Christian and human rights-based moralities arouse heated discussions relating to SRH services [17], despite both being external to the local context and by-products of modernity and globalization [18]. However, delaying the incidence of teenage pregnancy and early childbearing remains a priority to ensure the prevention and management of associated negative consequences of early pregnancy such as maternal mortality, morbidity, unsafe abortion, low birth-weight and infant mortality rates [13, 14].

Studies have noted how competing discourses relate with each other, particularly, global discourses interacting with and shaped by local socio-political contexts [19], competing for dominance or indeed existing in plurality or hybridity [19–21], and sometimes perpetuating the lack of access to information and services for young people [22]. For this paper, a critical postcolonial perspective is drawn upon to understand the discourses that surround the increasing burden of adolescent fertility, highlighting the structural transformations that have impacted negatively on young people and their agency [23, 24]; in this case, the ability to make fertility-control-related decisions. Epistemologically, the postcolonial critical perspective highlights the linkages among the domains of human experience—the psychological, ideological, social, political, intellectual, and aesthetic, in ways that show how inseparable they are [25], and their on-going manifestations in health and wellness of communities [26].

While post-colonialism more predominantly unearths inequalities associated with race and class, it can also be used to understand matters pertaining to religion, cultural beliefs, identity, double consciousness and, hybridity [27, 28]. This perspective posits the creation of pervasive colonial subjects with a “double consciousness or double vision”; where persons who live in former colonies develop dissonant cultures, values and beliefs, which allow them to view the world in a pluralistic way. To understand further the circumstances that reinforce high adolescent fertility in Zambia, this paper explores the multiple discourses or “multiple consciousnesses” that concurrently shape adolescent fertility in Zambia, drawing on a perspective that highlights the inseparable nature of dissonant views within the same people; keeping in mind the reduced access to SRH services among young people [25] and their pursuit for more power over their SRH decisions.

## Methods

### Study design

A case study design [29] was adopted for the study, using qualitative methods. This design allowed an in-depth exploration of adolescent fertility in Zambia.

### Study site

The study was set in Lusaka province [30]. Data were collected from Lusaka and Chongwe districts [30] from January 2018 to July 2019. The sites were purposefully selected for this study with Lusaka representing a more urban setting while Chongwe represented a rural and peri-urban setting. Lusaka district houses Lusaka, the capital city of Zambia. Lusaka city is situated in the Central Part of Lusaka Province and the country, with a population of 1,747,152 people. While the natural vegetation has been cleared due to urbanization, the open deciduous woodlands account for 80% of the forested areas. Economically, Lusaka is the second-largest economic centre of Zambia, notable for the diversification of production of goods and services. The city is the centre for national social amenities and most governmental and non-governmental department headquarters. The district is coordinated by the District Administration, which is made up of the Council, Office of the District Commissioner and the traditional administration [63].

Chongwe district is about 40 min away from Lusaka City. It has a population of about 182,174 people, of whom, 89,265 are female and 92,909 are male. The population density is 22.2 persons per square kilometre. Other than the Game Management Areas, Chongwe has three major vegetation types—Dry Miombo, Mopane and Savannah Woodlands, but suffers heavy deforestation due to charcoal burning, farming and other activities. This is not surprising as Chongwe is predominantly agricultural, with agricultural activities in crop production, horticulture and livestock production as the main sources of income. The district is run by the District Administration, which is made up of the Council, Office of the District Commissioner and the traditional administration under the leadership of Senior Chieftainess Nkomeshya Mukamambo II of the Soli people [63].

Both the rural and urban perspectives were captured in the study, with the inclusion of the more peri-urban views from both Chongwe and Lusaka. The Zambia Population HIV Impact Assessment (ZAMPHIA) survey showed a reduction in HIV prevalence from 13.3% in 2014 to 11.6% in 2016 [64]. Despite a high disease burden, much progress, such as the reduction in HIV prevalence rates have been made, in addition to other declining indicators such as Maternal Mortality Ratio (591/100,000 live births in 2007 to 278/100.000 in 2018), Infant Mortality Rate (70/1,000 live births in 2007 to 42/1,000 live births in 2018) [30]. Despite a record reduction in fertility within the reproductive age group from 6.1 in 1996 to 4.7 in 2018, teenage pregnancy has remained stable and constant over the last 15 years, indicating high adolescent fertility in Zambia [32].

### Study population and sampling and sample size

Discussions were held in purposefully selected locations in community schools and high schools. Interviews with other stakeholders were conducted in government ministries, schools, churches, clinics, and homes. The study included a total of 25 individual interviews adolescents (15–19) and 8 Focus-Group Discussions (FGDs) of 5–8 participants were conducted in selected locations in both rural and urban settings, at community and high schools (Table 1).

**Table 1:** Characteristics of focus-group discussion participants

Focus-group discussions	Number of FGDs	Lusaka	Chongwe	Age group	No. of participants
*Male adolescents*	3	–	–	15–19	–
Male	–	1	–	15–19	8
Male	–	–	1	15–19	8
Male	–	–	1	15–17	6
*Female adolescents*	4	–	–	15–19	–
Female	–	–	1	17–19	6
Female	–	1	–	15–19	8
Female	–	1	–	15–19	8
Female	–	1	–	15–19	8
*Male & female*	1	1	0	15–19	8
*Parents—mothers*	1	1	0	35–59	7
Total FGDs	9	6	3	–	67

Also, 8 key informants were interviewed: 3 from government ministries, alongside 2 teachers, 1 religious leader, 1 health worker, and 1 parent (Table 2). All data were collected from Chongwe and Lusaka districts. An FGD with mothers was also done.

**Table 2:** Characteristics of interview participants

	Interview participants	No. of interviews	Lusaka	Chongwe
1	*Interviews with adolescents*			
	Male	6	2	4
	Female	19	3	16
	Total	25	5	20
2	*Key-informant interviews*			
	Government ministries	3	3	0
	Parents—father	1	1	0
	Teachers	2	1	1
	Health workers	1	1	0
	Religious leader	1	1	0
	Total	8	7	1
	Total interviews	33	12	21

Purposeful sampling was employed, with snowball sampling also incorporated where there was a need for more information from participants who were not initially included in the study proposal but were additional sources of information needed to meet the study objectives. Community health workers were engaged in the selection of adolescents in Chongwe districts. These adolescents lived in the community around a community school in the district. Adolescents in Lusaka were selected by teachers at the schools that were selected. Recruitment of key informants was done via snowball or purposive sampling too. The sampling method was based on knowledge of informants included in the study. All the study participants were not economically independent as they lived with either a parent or guardian. While a few had completed secondary school, majority of the adolescents were still in school-ranging from grade 8 to grade 12 (junior and senior secondary school).

### Data collection

Data was collected through individual interviews and FGDs, steered by discussion guides (see Additional file 1). For the adolescents, trial interviews were done first. A total of 18 interviews were conducted with adolescent boys and girls, exploring the social construction of adolescent fertility. The tools were then adjusted, for the critical perspectives that emerged from the data, using both the shared meanings and the various relationships that may reduce their access to information and services. After that, the discussion guides were revised, and more data was collected using the revised tools; to adequately document adolescents’ experiences and perceptions of fertility control in Zambia. Also, notes were taken from participants who did not consent to be recorded. Other than the formally organized discussions, additional informal discussions with various informants were done throughout the study data collection period—January 2018 to July 2019.  

### Data analysis

All the audio-taped data was transcribed verbatim, and the transcripts were imported into NVivo 10 [31] for management and analysis. Initially, thematic analysis was used to analyze the data [32]. Sense-making was iterative-started during data collection through revision of tools, more data collection, translation and transcription. Critical Discourse Analysis [33] tenets were applied, drawing on social constructionist and critical perspectives to make sense of the data. Fairclough posits that discourse shapes our understanding of reality, highlighting how divergent views expressed by different actors in society can shape behaviour [33]. We started by exploring how fertility was constructed by adolescents and how the different moral influencers shaped these shared understandings. Thereafter we identified the competing discourses that influenced these shared understandings, from a postcolonial critical perspective [25].

### Ethical considerations

Ethical approval was sought from Excellence in Research Ethics IRB (REF 2017-Apr-007). Informed consent was sought from all the participants before the interviews were conducted. Participation was voluntary, non-remunerable, and the researchers sought consent to record the discussions separately from consent to take part in the study. Consent from all adolescents who assented was sought from their parents or guardians, and permissions to conduct interviews were provided by the relevant authorities. Confidentiality was upheld throughout the study.

## Results

Discussions revealed that the age of adolescents, marital status and marital norms, Christian values, and rights-based values influenced adolescent fertility control. In this section, we present findings according to 4 main themes: (1) age and fertility control, (2) marriage, contraception use and fertility control, (3) Christianity and fertility control and finally, (4) health, rights and fertility control.

### Age and fertility control

On the one hand, adolescents believed in their parents’ advice, including how to control their fertility. On the other hand, the parents also felt it was their duty to advise their children on what to do generally, including how to control their fertility. The basis for this belief was that parents had been through similar experiences when they were adolescents themselves. When a guidance and counselling teacher was asked what she would say to her daughter if she found out she was sexually active, this is what she said:

“(…) look at your age. You are still very young. Why can't you wait until such a time that society (…) [can accept your decision] or you've completed your education, then we bless you? You get married in there right away. You go and start your home. Why can't you wait until such a time? When you rush my daughter, you crash…always. Follow what the elders say because they have been through it. Abstain from sex. You can enjoy [sex] for maybe one or two minutes, but after that, what happens? You become pregnant and this is what leads to (…) early pregnancy. When you are not ready to have a child, you have it. Why? Because you have indulged in unprotected sex.” (Interview- Teacher- Guidance and Counselling; Christian and Parent)

Since information from parents came in the form of advice, or guidance, the young people were compelled to obey because they trusted their parents. Adolescents were supposed to follow this advice, citing their obligation to listen to parents and other adults in general, because they ‘knew better’ and had been through all those experiences before. Besides, it was overall viewed as respectful. An adolescent girl who was asked why she listens to her mothers’ advice said; *“(…) it’s having faith because they have been through life; she knows all things (…) (Adolescent girl, 18 years, Completed school, Chongwe).*

However, the discomfort of the sex and fertility control topic between parents and their children also shaped how information or advice was packaged. The young people were unable to find out more from their parents due to the instructional nature of the information. An adolescent boy had this to say, “*Guardians are very restrictive people and can never allow their child to start involving themselves into sexual activities when they are young*”. *(R3, FGD, Boys, 15 to 19. In-School, Lusaka).*

While young people could trust their parents’ advice, they also felt the unmet need to make their own decisions and that their parents did not understand their experiences adequately, with the absence of an opportunity to address these unmet needs, or to have discussions rather than receive instructions. They thus opted for other sources such as other family members and peers. Despite the need for more information from their parents, the adolescents were expected to obey their parents’ advice, as ‘well-mannered’ children should.

“We don’t talk to our parents about this because our parents are very much strict when it comes to these things. We fear that if they know that we are found with condoms, they will start thinking that we are involving ourselves in bad things.”

(R8 FGD, Boys, 15 to 19. In-School, Lusaka)

Parents also felt obligated to talk to their children about fertility control, but it was also generally felt that fertility control discussions were beyond them, to an extent. They strongly contended against many other information sources, including what was termed as ‘bad information’ that the young people were exposed to from peers, entertainment, and social media. In the end, parents felt that young people choose their influencers, and they hoped that their decisions would not lead to negative outcomes such as unintended pregnancy.

Respondent 1: (…) this is how these children are, even if you discipline them, they don’t behave.
Interviewer: She really disappointed you?
Respondent 2: (…) she was just saying that we do discipline children, it’s just that others don’t behave no matter how much effort you put to discipline them.
Respondent 1: Others do listen when an elderly person gives them advice; they have even completed school but some children, never listen [fall pregnant]. (FGD, Mothers to adolescents, Lusaka)

While most parents were expected to provide information to the young people, information and guidance were exerted beyond parental relationships. Most of the other older people in the communities also provided ‘guidance and advice’ to adolescents, and this was widely accepted. Also, the community played a role in influencing the fertility control decisions of the young people, by sharing their opinions. Adolescents felt watched and continuously monitored by their communities.

“(…) if people know that you are abstaining in your community they will really accept you and you will be an example for other girls who don’t practice abstinence. Parents (including other adults in the community) will be like, have you seen that child, she has good behaviour and she completed school and she doesn’t do bad things, you have to be like her.” (R3, FGD, Girls, 15 to 19. In School-Afternoon, Lusaka)

Keeping in mind this view of adolescents by older people in general, the packaging of information was also grounded in the influencers’ varying opinions and values; traditional marriage values, Christian values and rights-based values. It was common to find teachers and health workers who were faced with conflicting roles; since they were also parents to adolescents themselves. For instance, all teachers were tasked to provide SRH information to young people, regardless of the subjects that they taught. However, this role also interacted with how they packaged information for their children if they had any or other roles they played in the community. While SRH information is provided in schools, how it was implemented was impacted by the values and beliefs of teachers or health workers.

“(…) so you are talking about encouraging them to use SRH services or to cope up (stay abstinent). As a parent, the right time for them is when they have grown up and can stand on their own. Then, as a guidance counsellor, if I meet my daughter saying things like that, I can tell her not to engage herself in these sexual relationships before her right time, if anything, she should focus on her education. That is in my path as a parent. Focus on your education. Do not ever engage in any sexual relationships either at home or school (…) especially girl children should not start indulging into sexual relationships because once they start their attention in class will be disturbed. You know they are just sexual relationships [mere sexual relationships]. They go with it and a lot of things, negative things can happen. Once they start they know that, okay, this is what I will get and you know as they continue having sexual relationships, problems begin (…)” (Interview- Teacher- Guidance and Counselling; Christian and Parent)

### Marriage, contraception use, and fertility control

While discussions against adolescents’ access to services cited the right time that access would be acceptable: when they were older, financially stable, or married, some exceptions were noted for adolescents who were married. This is because marriage was associated with being independent, and the most justifiable reason why a person was supposed to access contraception. It was generally expected and acceptable to engage in sexual activity and to have children when married. They linked contraception to a later time when they were married and stable, capable of starting a family. When asked if adolescents were too young to use contraception and when the right time to use contraception was, an adolescent said:

“I think the right time to use contraception (…) it is when you feel like uh when you see that your life is stable (…) Yes, that is the right time I think to use contraceptives. Maybe when you are married and you have your own home and you have your own house.” (R3, FGD, Girls, 16 to 17. In and Out of School, Chongwe)

However, it also meant that in-between adolescence and marriage, adolescents were not supposed to use contraception, leaving them vulnerable to teenage pregnancy. However, having had a child increased autonomy over their lives and the expectations from the health workers and the community for the young people to access contraception information and services. In the rural areas, marriage was outright seen as an empowerment tool for not only the young person but for her family as well. Marriage or having a child allowed the adolescent to access contraception, and to also be treated as an adult in the community.

“There are those like young girls in the villages where they have no option rather than getting married (not socio-economically empowered). They just take it like, in villages, a girl is a source of income, and so like if she gets married, they will get some money so the girl will be forced to get married by the parents (…)” (R2, FGD, Girls, 15 to 19. In and Out of School, Chongwe)

### Christianity and fertility control

Adolescent fertility control discussions were also strongly influenced by Christian beliefs. Over 98% of Zambians are Christians [8]. While marriage qualified access to fertility control information and services, sex before marriage was viewed as a sin, as most of the adolescents had heard this from their homes, their other relatives, from church and in schools. During a discussion about deciding to engage or not engage in sex, and when asked why she felt it was expected of her, in particular, to be abstinent, an adolescent girl said:

“Because of my tradition first of all. I feel like as Zambians, we are not expected to have sex until we are married and also because I am Adventist [Christian denomination] so it’s like [that means] sex before marriage isn’t a good thing at all. (Adolescent girl, 18 years, Christian, Completed secondary school, Lusaka)

Christian views of abortion as a sin, especially for adolescents, were very common among the discussants. The magnitude of the sin of abortion was great because it was viewed as murder in the wake of an existing sin-engaging in sex before marriage. This level of sinfulness and the murder made having an abortion upsetting to the adolescent, and the rest of the community, primarily since abortions were negatively associated with witchcraft or wizardry. When asked about situations when abortions take place, an adolescent girl said:

“Abortion mostly is done when like you get pregnant by mistake. Mostly you find a situation whereby you are pregnant and you are not ready to keep that child. So people go to the clinic to abort and kill the child, but it’s very bad. As Christian, we are not supposed to do that”. (R10, FGD, Girls, 15 to 19. In School-Afternoon, Lusaka)

It was only acceptable to practice abstinence. Moreover, failure to do so left the adolescents with multiple explanations for their ‘bad’ behaviour. When asked if young people could abstain, an adolescent boy said;

“It is possible because we can’t say us as Christians we cannot abstain. You don’t have to be committing adultery, so it is possible to abstain.” (R8, FGD, Boys, 15 to 19, In School, Lusaka).

In another discussion with adolescent girls, an adolescent said:

Facilitator: Ok, what is the reaction that our community makes when they see a young person who is not abstaining from sex?
Respondent 5: They criticize the person who does not abstain and they a lot of bad things about the person who cannot abstain. They even start talking about parents that they did not teach her properly and things like that even when parents are not the causes of that (FGD, Girls, 15 to 19. In School-Morning, Lusaka)

It was noteworthy that during the discussions with the young people, there was also a tendency for some discussants to justify their own opinions with religious messages attached to them. When this happened, it was also not very easy for the discussants to question this opinion. As such, religiosity attached to opinion was more acceptable and more dominant than any other discourse or opinion related to adolescent fertility. For instance, asked whether young people should receive SRH information or not one adolescent said;

“I think in most of the time we lack knowledge because even in the bible it is written my people perish because of lack of knowledge. I think the best way to avoid pregnancies and abortions is about awareness. I think there should be a policy where like people should be visited in their homes to learn about these things.” (R7 FGD, Girls, 15 to 19, In School-Morning, Lusaka)

Another adolescent said:

“I think the best thing is to abstain. Even in the bible, it is written in books Ecclesiastes that do whatever you do in the time of your youth but remember you will be judged alone on what you do. So you have to abstain.” (R3, FGD, Girls, 15 to 19, In School-Morning, Lusaka)

Adolescents who shared their opinions regarding what is right or wrong, according to the bible or concerning God, were less likely to be questioned or opposed compared to those that did not speak with this religious authority. When religion was attached to service provision, adolescents were not supposed to receive services because they were not supposed to be sexually active before marriage.

While the religious discourse was persuasive at the individual and community level, it was present and dominant structurally too, since Zambia was declared a Christian nation in the constitution. The Ministry of National Guidance and Religious Affairs, MNGRA held the mandate of enforcing national values and operationalizing the declaration in the country; indicating the influence of Christian values at a national policy level. National values included: morality and ethics, patriotism, democracy, dignity (inclusion), governance, and development. Despite the strong religious emphasis mainstreamed by the MNGRA, it was also noted that inclusion and dignity were highlighted in the national values. Therefore, it was noted as essential to consider the health needs of young people, with particular consideration for their specific needs. There was also an emphasis on promoting these values within the smallest unit that the young people were part of (family or home) and the need to extend those values at that community and national levels as well. When asked about how to package messages for young people, a policy implementer said:

“(…) from the perspective of the ministry [MNGRA] (…) the basis is the family. It starts with the family, then we have a community and then we have a society. So when it comes to talking about such issues [SRH], our emphasis is that it has to start from the family. The parents should be able to talk to their children about some of these issues before they go to school. Because everything we are doing (…) it starts with the family. [Policy Implementer, MNGRA, Lusaka]

### Health, rights and fertility control

Few adolescents (and from Lusaka district) viewed access to contraception and abortion as a right; in that, they felt they needed to know about contraception and abortion for them to make better decisions.

Facilitator: I am going to read a statement to you guys and you have to tell me how you feel about this statement, “Adolescents are too young to know about contraceptives. The right time to talk about these things will come in future when they are older.” How do you feel about this statement?
Respondent 1: For me, I feel it’s wrong because we have to be learning about things. If we don’t learn now, we will be ignorant about these things so if we learn, it will help us know them. Most teenagers are having sex anyhow so I feel we have to learn about these things.
Interviewer: So you feel that everyone has to know about contraceptives and family planning?
Respondent 1: Yes
Interviewer: Anyone else?
Respondent 7: For me… I would say we need to know about family planning because it will give us self-control and let us know where we are supposed to stand and give us dignity…on what we should and what we should not do.” (FGD, Girls, 15 to 19, In School-Morning, Lusaka)

The Ministry of Health, MoH, noted that young people had the right to receive SRH information and services. Also, it was noted that efforts to provide adolescent-friendly services were made, but had been primarily reactive; focusing on the sexually-active adolescents, rather than all the adolescents. When asked about the health sector response to adolescent fertility, a health policymaker said:

“(…) it depends on who is supporting adolescent health programs… but as a ministry [MoH], we have realized that our messaging in the recent past has focused more on the sexually-active adolescents who are only 38% of the adolescents. According to the Zambia Demographic and Health Survey of 2014, 32% of adolescents are sexually active, meaning 62% (…) are not sexually active. So we have tried (…) to become more comprehensive so that we do not leave out the majority 62% [abstinent adolescents]. So our new approach is to make our messaging more comprehensive and to even re-orient our adolescent health spaces (…)” Policy maker-Adolescent Health, Ministry of Health, Lusaka.

The young people also acknowledged that the services were available in some settings. It was noted, however, that there was a focus on those who were pregnant or sick rather than all the adolescents who went to the health facilities. Also, adolescents who tried to access service from health facilities felt that there was an insufficient response for them without any form of adversity linked to their quest for SRH services. An adolescent girl said;

“Services are offered but these people who work in clinics are not serious. All they care about are just sick people because when I am watching T.V., I see other countries like in America they are very serious about these things but in Zambia, it seems like we have got low services and they don’t pay attention. The only time they will concentrate on is when they take you that you are pregnant. So services are not really there” (R10, FGD, Girls, 15 to 19. In School-Afternoon, Lusaka)

More recently, the use of social media to disseminate information and engage with adolescents has been introduced. However, the need to increase demand and to reach those who are not accessible via these platforms was noted. Historically, the role of the MoH in ensuring youth-friendly services for adolescents has been emphasised. These services were provided with some support from the Ministry of General Education, MoGE through the provision of CSE in schools. While acknowledging that the services could be improved, the MoH emphasized that services were the last resort; an indication that the adolescent had not been reached before they sought medical care. Therefore, a shift in focus from the health services but rather to reach adolescents in their homes and communities was emphasized, to promote healthy behaviours rather than leaving the adolescents vulnerable to early pregnancy or diseases.

## Discussion

In an attempt to understand the competing discourses that shape adolescent fertility in Zambia, our findings highlight the influence of marital status and related norms, Christian, and SRH rights-based values, all navigating the nonautonomous and disenfranchised place of adolescents in their communities. While the divergent nature and characterization of the adolescent fertility discourses was evident, we argue that the multiple discourses concurrently and dissonantly shape adolescent fertility, compounded by adolescents’ lack of agency in fertility control decision making, maintaining the restricted access to SRH information and services and related negative adolescent SRH outcomes.

In this study, there was a consensus on the restriction of adolescents from access to fertility control information and services. In most cases, the Christian-moral values, usually attached to marital norms, prohibited adolescents from engaging in sex before marriage and consequently restricting their access to fertility control. However, with the urgency to reduce the health risks associated with the unmet need for fertility control—the effects of early childbearing and marriage, access restrictions were reconsidered. The findings indicate that on the surface, the adolescent fertility discussions seemed clichéd as all the discussants referred to- the sin of sex before marriage and the need for young people to wait for marriage to be able to control their fertility without barriers. Also, adolescents severely lacked autonomy particularly regarding the control of their fertility. At the same time, being married (early marriage) or having given birth before (early childbearing) provided sufficient agency to access SRH services with fewer barriers.

While agency alone is a complex concept [23], guidance on fertility control decisions was generally accepted by both the young people and their fertility control influencers—parents, teachers, health workers, and policymakers. The lack of agency was more apparent through adolescents’ lack of financial independence, reduced decision-making power to utilize SHR information and services [23], and this typically situated them within the brackets of a marginalized or socially secluded group [34]. All the discussants seemed to be at peace with the adolescents’ lack of autonomy as it was culturally acceptable for young people to be obedient without questioning the elderly, radicalizing the young people who sought to control their fertility. As other studies have shown [35], this was the case despite the discomfort of discussing SRH issues with the elderly, especially their parents.

Adolescents’ lack of autonomy and their subsequent fertility control-related experiences of marginalization contest the classic politics of seclusion [34] as adolescents are situated in a transition phase that all the older community members had experienced before; giving the influencers a ‘shared experience’ of adolescence and fertility. Having been adolescents themselves, parents, teachers, and other community members felt that they were better placed to make decisions for young people, who were also uncertain due to their novel experiences of adolescence and the fertility control decisions. A member of their community, especially their parents, teachers, and health workers had increased authority over their fertility-control decision-making due to their age. In contrast, adolescents experienced reduced agency to actively access desired information and services due to a lack of sufficient SRH knowledge.

In addition, while a higher socio-economic status (higher educational attainment and wealth status) has been linked to reduced fertility in Zambia [11], adolescents without access to education and with low socioeconomic agency were most vulnerable to early marriage and childbearing- usually seeking to secure financial and material support from sexual relationships and marriage [36]. A study exploring the agency of adolescent girls in India showed that life-skills training boosted self-esteem and self-confidence in adolescent girls increasing their ability to make decisions for themselves. Also, their value at the household-level was increased, thereby increasing their ability to negotiate their preferences [24]. Disease-specific programs in sub-Saharan Africa such as the DREAMS (Determined, Resilient, Empowered, AIDS-free, Mentored and Safe), a comprehensive approach to preventing HIV among adolescent girls and young women (AGYW), have sought to address structural barriers beyond the health sector, increasing the agency of AGYW [37].

However, other significant sources of agency related to fertility control must also be considered, in this case, early child-bearing and early-marriage. Noteworthy changes in how young people were treated occurred, and the information and services they could access after marriage or giving birth, despite their age. Also, the health sector provided a reactive approach to meeting adolescent fertility-control needs. The response was mostly after the young people had conceived or contracted an illness and they were undoubtedly in need of SRH services at facilities. Besides, visiting the health facility while healthy and ‘merely’ seeking information and guidance seemed problematic for young people. Such findings justify adolescent SRH interventions that seek to increase demand for information and services [38].

Adolescents also reported less judgment from the community for married adolescents, especially in the rural areas where early marriages were rampant. Understanding that early marriage and child-bearing have significant impacts on increasing adolescents’ agency [14] could explain the stability of teenage pregnancy in Zambia over the years, despite interventions aimed at providing more responsive services. Also, restrictions on fertility control decision-making among majority unmarried and nulliparous adolescents increase the risk of unsafe abortion from clandestine sources [13]; a situation worsened by poverty, unequal gender norms, corruption, poor service provision [39] and religious teachings which compelled adolescents to conceal sexual activity rather than avoid it. In addition, the value and emphasis of education over marriage increases the likelihood of ‘unintended’ pregnancy among young girls [40], though Sedgh et al., 2015 have argued that the majority of teenage pregnancies in developing regions may indeed be intended due to the norms of early marriage and early child bearing [41].

Adolescent fertility interventions have been more focused on expanding access to services [35, 42]. However, situating the increasing burden of adolescent fertility and associated socio-economic risks within the historical and social context unearths underlying multidimensional barriers that continue to hinder the success of interventions that are targeted towards reducing adolescents’ fertility, in line with what Tyson 2014 asserts, while we attempt to understand adolescent fertility in more apparent ways, in this case, high adolescent fertility or early child-bearing, [25] understanding personal level conflicts and how they interact with historically developed ideologies and identities may help to understand phenomena more clearly.

In this study, the dominant moral viewpoints; marital status, Christian-religiosity, and SRHR influenced adolescent fertility discussions, despite their ideological differences. Historically, early marriage has been acceptable in many traditions in Zambia, with preparations for marriage and child-bearing beginning at menarche. Despite a global shift away from early marriage, with more focus on delaying the onset of child-bearing and attaining more years in school, adolescents, especially in rural areas and in the urban poor areas [7] continue to have higher fertility. Critical messages for young people resonate with delaying the onset of child-bearing [5]. As they develop the capacity to claim their right to health, adolescents still face stigma or messages that detour them from seeking information and care from trusted sources. A potential response to such barriers would be the implementation of community-based interventions that address judgmental attitudes towards unmarried adolescents who seek fertility control [43, 44]. To be successful, such interventions would, among other strategies, focus on addressing intergenerational barriers to discussing sexual and reproductive health matters in the community [43].

On the one hand, we assert the importance of increased autonomy in adolescent fertility-control decision-making, the devastating effects of the lack of it, noting the unfavourable circumstances that facilitate autonomy among adolescents. On the other hand, the major discourses that impact adolescent fertility were somewhat divergent in guiding adolescents on fertility control. The teachers and the health workers sometimes found themselves in the dilemma of providing competing messages, because of their other roles in the community, such as being parents. Also, the influencers themselves did not necessarily identify entirely with one ‘moral’ viewpoint, but with multiple moral viewpoints at a time.

In Zambia, globally-framed human rights and Christian-religiosity have had a history of divergence in shaping moral sensibilities, including health [18]. As such, playing different roles in the community and not necessarily picking one moral viewpoint over the other also meant that fertility-control influencers themselves experienced dissonance with much of the information they were providing to young people. A study exploring the implementation of Comprehensive Sexuality Education, CSE in Zambia showed that teachers, amid moral dilemmas about the content of the curriculum exercised paternalism as they used their discretionary power to alter the messages to protect young people if they felt the need to [45].

With the health sector spearheading the empowerment of adolescents through sensitization about their right to SRH services through policies and legislation, expectations for the frontline civil servants such as teachers and the health workers to implement these strategies was high, and they did. However, the parents, teachers, health workers or policymakers carried with them an ‘identity-crisis’—a view that young people had the right to access the SRH services within the global health discourse. Conversely, they too shared traditional marital norms that potentially allowed young people more decision-making power or Christian values that compelled them to advise young people to avoid sexual activity altogether until marriage.

This complex, or “unhomeliness” as Tyson (2014) outlines is likened to being “in a home away from home’ but highlights the confusion that most influencers have and share with adolescents regarding fertility control and more generally, access to SRH information and services. Despite active decolonization attempts spanning from 1964 (independence), the identity crisis or dissonance of competing values among and within different influencers of adolescent fertility control decisions continue to shape pluralistic viewpoints, which were manifest among adolescents when discussing fertility. In this paper, the discussions with adolescents and other informants in the community, are familiar, but using a critical lens reveals the thriving confusion people, as “multiple consciousnesses” and we argue that these competing consciousnesses contribute to the high and unchanging fertility levels among young people in Zambia.

## Limitations and strengths

This study aimed to understand adolescent fertility in Zambia. The need to broaden the study sites and to deepen the discussion with a gendered lens to highlight unique access and utilization inequalities is noted, and could be done in subsequent studies. In addition, the paper only highlights three competing discourses, based on the discussions with the study participants and only  religious views from the Christian sect were captured as they were the most dominant in the discussions. Despite collecting data in only two districts and not representative of the whole of Zambia, the study explored the shared understandings of adolescent fertility which may be transferable in relation to other studies undertaken in Zambia on adolescent fertility; adding to the credibility of the findings for many settings in Zambia and beyond [46]. Moreover, while most adolescents were interviewed in this study, the insights drawn on were based on additional sources; teachers, parents, health workers and health policymakers, providing a variety of data sources. Despite sample size limitations, different data collection methods were also employed; formal and informal individual interviews, FGDs and document reviews [47]. Shenton, 2004 highlights the necessity of adopting research methods that are well established: specific procedures that have been used before [47]. This was a strength in this study as the data collection processes were well adhered to and explained in the methods section. Lastly, the data analysis and interpretation was an iterative process; therefore, not based on a single subjective interpretation [47].

## Conclusion

Amid their quest for more autonomy around decision-making in general, adolescents highlighted competing discourses that impact their understanding of contraception, abortion and abstinence, and these were shaped by various values in the institution of marriage, Christianity and also a global SRHR discourse. It was generally understood that adolescents lacked agency; the agency to make decisions about their fertility and generally their sexual and reproductive health. This lack of agency had its foundations in their reduced social and economic empowerment. The disenfranchised view of adolescents was also a driver of the continued structural seclusion from fertility control decision-making capabilities. Tapping into a social exclusion discourse, the young people faced a unique form of seclusion based on the fact they did not belong to a closed group in another setting, per se, but belonged to a group that every adult around them had belonged to as well. This view of young people, therefore, kept all the various actors interested in making the “right’ decisions for them, until they had the full autonomy to do so- later in life. Also, while actors provided advice to the young people, this advice came in very varied forms as it was shaped by the various moral worlds they upheld, upheld the most, or at a particular time, or in a particular location.

Keeping in mind the double consciousness concept and how it allows the different moral worlds to impact adolescents’ influencers, we argue therefore that the competing dominant discourses or “*multiple-consciousnesses*” that moral world influencers draw on send mixed messages to young people and they perpetuate the deteriorating or consistently undesirable fertility indicators among adolescents. While a health worker advocates for SRH uptake at the health facility, the same health worker at home becomes a mother who does not allow the adolescent to access these services or the church member who teaches that sex before marriage is a sin. These “*multiple-consciousnesses*” continue to impact negatively on interventions aimed at addressing adolescent fertility. A complete shift or transformation and alignment among the different moral influencers in their collective conceptual orientation of the gendered SRH needs of young people and how to address these needs sustainably would provide an appropriate narrative of advice across moral influencers. Also, multi-sectoral collaboration of various line government ministries creating a basic package for young people—for example, socioeconomic empowerment activities such as life skills training while encouraging girls to stay in school, with the provision of youth-friendly health services.

## Supplementary Information

**Additional file 1.** Discussion guide for adolescents.

## Data Availability

The datasets used and analyzed during the current study are available from the corresponding author on reasonable request.

## Abbreviations

SRH: Sexual and reproductive health
SRHR: Sexual and reproductive health rights
CSE: Comprehensive sexuality education
AYGW: Adolescent girls and young women
