# Supplementary material for: A critical discourse analysis of adolescent fertility in Zambia: a postcolonial perspective
Source: Reprod Health. 2021 Apr 6;18:75. doi: 10.1186/s12978-021-01093-z (PMC8022403; doi:10.1186/s12978-021-01093-z)
Supplement: Supplementary file 1 — Additional file 1. Discussion guide for adolescents. [file 12978_2021_1093_MOESM1_ESM.docx]

**Exploring moral worlds that shape adolescent fertility control strategies in Zambia**

*Sample Interview Questions*

# **Introduction**

- Tell me about yourself (age, upbringing, schooling, environment- rural urban, raised by parents or guardian, siblings, …)

# **Knowledge**

Studies have shown that adolescents do not have sufficient information about SRH issues including family planning and contraception (reference). Knowledge about contraception among adolescents is vital for them to make decisions about what methods are most appropriate for them as well as where to access them. The ZDHS provides information on knowledge of contraception among adolescents aged 15 to 19 (95%) capturing knowledge of at least one method. However, this knowledge level is very problematic and needs to be explored further because most studies on adolescents point to the fact that adolescents have low knowledge levels. In addition, the questions are phrased for women who are married in a way presupposing that those who use contraception and family planning should always be married. This way of introducing the questions may increase the problematic nature of the findings on knowledge of contraception.

- What do you know about fertility control (ways of preventing o dealing with pregnancy), in your opinion? What is its importance? Irrelevance? (probe for fertility control methods)
- What is contraception/ family planning, in your opinion? What is its importance? Irrelevance?
- “Adolescents are too young to know about contraception/ the right time to talk about it will come later in the future when they are older”. What do you think/ how do you feel about these statements?
- Who do you talk to about (fertility control) contraception/ contraceptives/ abortion
- How do you feel/ what do you think about the information you receive / have received/ have been receiving is adequate?
- What do you know about the services being offered to young people at the health facilities? Ever accessed?
- What do you think your parents/teachers/health workers/church leaders think about you or your friends using contraception?

# **Abstinence**

# Major campaigns to reach adolescents have been focused on abstinence as the main message adolescents need to hear. Almost all moral worlds seem to resound this message when “advising” adolescents about fertility control and abortion. Information on how adolescents understand abstinence needs to be checked in order to see the value of this information. Other than those adolescents abstaining after birth, (9.8%), the ZDHS captures abstinence as “adolescents aged 15-24 who have never had sexual intercourse, and have never-married.

#

- - What know about the term abstinence? What does the term abstinence mean to you?
  - “Abstinence, ili che!” What do you think of this slogan? What does it mean to you? Symbolize?
  - What you think is the importance of abstinence in your community? How important is abstaining to you?
  - Who should abstain? (Young people, older people, married, unmarried…) Is abstinence possible?
  - What happens to someone who cannot abstain? How is the reaction from the community when a girl falls pregnant? If it is known than someone is abstaining, how do people take this?
  - How is abstinence looked at among young people compared to older people? Do you talk about it with your peers?
  - ‘Abstaining from sexual activity will make you more acceptable to people in your community”. Is this statement correct? Why?
  - Who in your opinion is expected to abstain more, girls or boys?
  - When do you thinks it is more possible to abstain, when one has money or when they do not?
  - “Adolescents should not be receiving fertility control information and services because they should be abstaining”. Would people in your community agree with this statement?
  - If adolescents had access to all contraception methods, would they still abstain? Give an example.
  - How do you feel when you manage to or fail to live up to this standard/ abstinence?
  - Do you think abstinence is the best option for you? Which is your best option?

# **Abortion**

Unsafe abortion is one key area surrounded by a culture of silence and discomfort, yet still continues being practiced, even among adolescent women. The ZDHS reports a prevalence of 1.6 among adolescents aged 15 to 19. Other estimates of abortion have reported that over 60 percent of women now at least one woman who has had an abortion. In addition, most records are hospital based indicating that they are only captured in case of a complication (15%). How then do adolescents understand abortion, and what is the best way to learn more about these understandings among adolescents?

- - What do you know about the term abortion? What does it mean to you?
  - What is your opinion about how right or wrong abortion is? What do people say about abortion in your community?
  - Have you ever heard of anyone in your community who ever terminated or tried to terminate a pregnancy? What happened?
  - What advice would you give to your colleague who wants to have an abortion? (Probe for special conditions? Provide scenarios) (the dominant views, and what do they think?)
  - Where would someone seeking abortion go?
  - What do you think about access to abortion? Do you think it is accessible to young people? Should they have access to safe abortion services?
